# Liaison Committee on Medical Education’s Diversity Standards and Medical School Attrition

**DOI:** 10.1001/jamahealthforum.2025.0697

**Published:** 2025-05-09

**Authors:** Mytien Nguyen, Tonya L. Fancher, Hyacinth R. C. Mason, Bassel M. Shanab, Shruthi Venkataraman, Sarwat I. Chaudhry, Mayur M. Desai, William McDade, Dowin Boatright

**Affiliations:** 1 Department of Immunobiology, Yale School of Medicine, New Haven, Connecticut; 2 Department of Emergency Medicine, New York University Grossman School of Medicine, New York, New York; 3 Division of General Internal Medicine, University of California Davis School of Medicine, Sacramento; 4 Office of Student Affairs and Department of Public Health and Community Medicine, Tufts University School of Medicine, Boston, Massachusetts; 5 Yale School of Medicine, New Haven, Connecticut; 6 Section of General Internal Medicine, Department of Medicine, Yale School of Medicine, New Haven, Connecticut; 7 Department of Chronic Disease Epidemiology, Yale School of Public Health, New Haven, Connecticut; 8 Accreditation Council for Graduate Medical Education, Chicago, Illinois

## Abstract

This cross-sectional study examines the association between the Liaison Committee on Medical Education’s diversity standards and medical school attrition demographics.

## Introduction

In 2009, the Liaison Committee on Medical Education (LCME) introduced diversity standards requiring MD-granting medical schools to promote recruitment and retention of students from diverse backgrounds, such as developing policies and programs to support and retain underrepresented students.^1^ Implementation of these diversity standards led to an increase in female, Asian, Black, and Hispanic matriculants to medical school.^2^ However, little is known about the association between LCME diversity standards and student retention.

## Methods

We examined attrition trends for MD students who matriculated in the 8 years before (2001-2008) and after (2009-2016) implementation. Because schools undergo accreditation at least every 8 years, the LCME would have evaluated all MD schools during these periods. Students self-reported sex, race, and ethnicity on the American Medical College Application Service. For the purposes of this study, underrepresented in medicine (URiM) was defined as Alaska Native, American Indian, Black, Hispanic, Native Hawaiian, or Pacific Islander individuals only. Students were categorized as having low income if their family income was $50 000 or less or reported receiving state/federal financial assistance. This study was deemed exempt by the Yale University Institutional Review Board because it contained nonidentifiable information. STROBE reporting guidelines were followed.

The main outcome is attrition, defined as withdrawal or dismissal for any reason. Attrition data were retrieved from the student record systems in January 2022. Annual trends in attrition before and after LCME implementation were assessed by interrupted time series analysis across sex, race, ethnicity, income, and their intersections using ordinary least squares regression with robust standard errors to adjust for autocorrelation. Statistical significance was defined by 2-sided *P* < .05. Statistical analyses were performed using STATA, version 18 (StataCorp). Data were analyzed from September to October 2024.

## Results

Between 2001 and 2016, 354 174 students matriculated into MD programs. After implementation of the LCME diversity standards, the overall annual trend in attrition rates decreased by a small but significant amount (2001-2008, −0.02% vs 2009-2016, −0.11%; *P* = .02).

**Figure 1.**
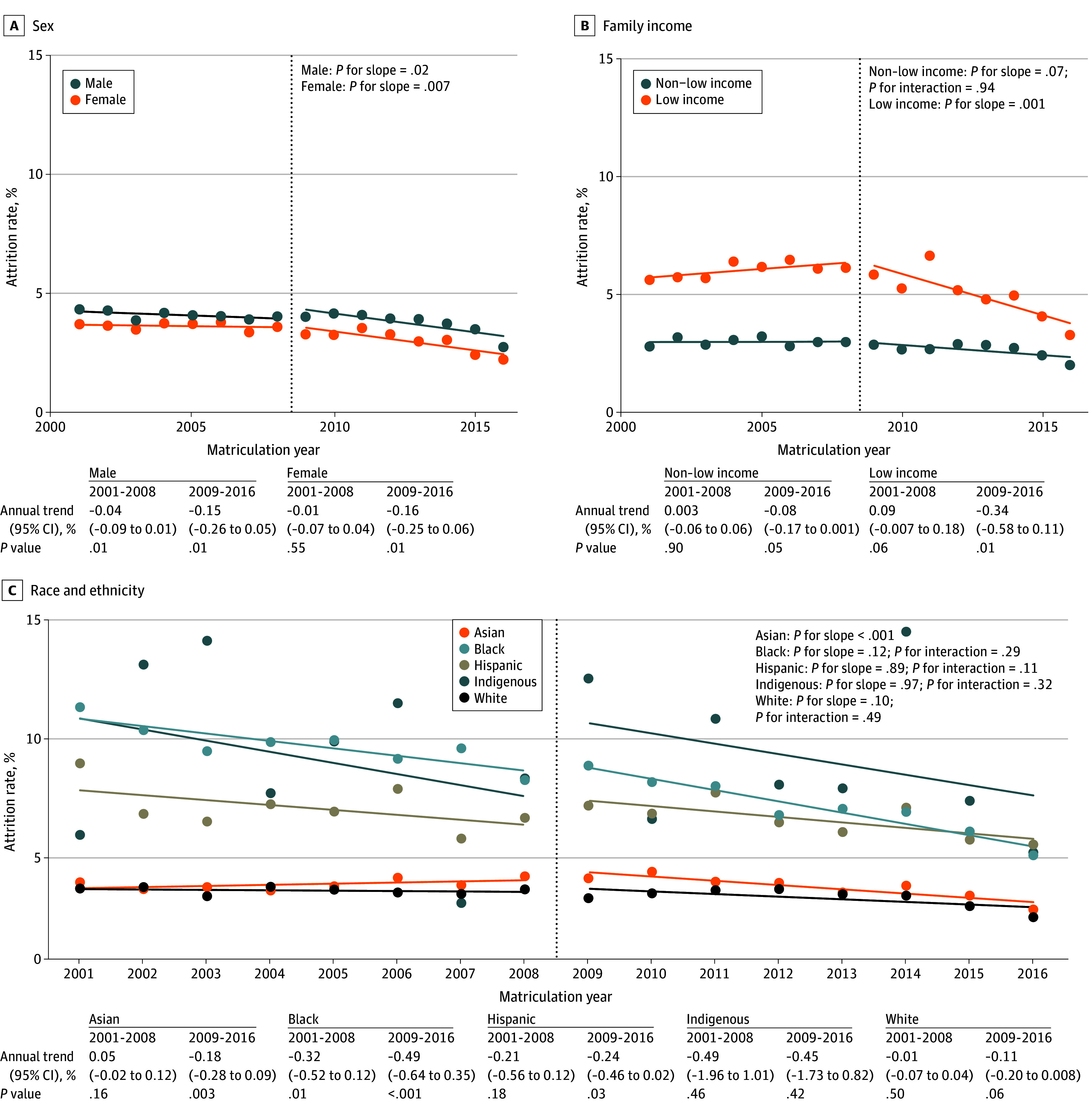
Attrition Rates by Matriculation Year Attrition rates for Doctor of Medicine matriculants from 2001 to 2016 by sex (A), family income (B), and race and ethnicity (C). Students self-reported sex, race, and ethnicity on the American Medical College Application Service. American Indian, Alaska Native, Native Hawaiian, and Pacific Islander individuals are grouped together as part of the broader Indigenous community. Dotted lines represent the line of best fit.

After LCME implementation, annual trend in attrition rates significantly decreased for female (2001-2008, 0.01% vs 2009-2016, −0.16%; *P* = .01) and male students (2001-2008, −0.04% vs 2009-2016, −0.15%; *P* = .02; Figure 1). Annual trend in attrition rates decreased significantly for Asian students (2001-2008, 0.05% vs 2009-2016, −0.18%; *P* < .001), with no significant change for other racial and ethnic groups. Annual trend in attrition rates decreased for low-income students (2001-2008, 0.09% vs 2009-2016, −0.34%; *P* = .001; Figure 1), with no significant change for non–low-income students.

**Figure 2.**
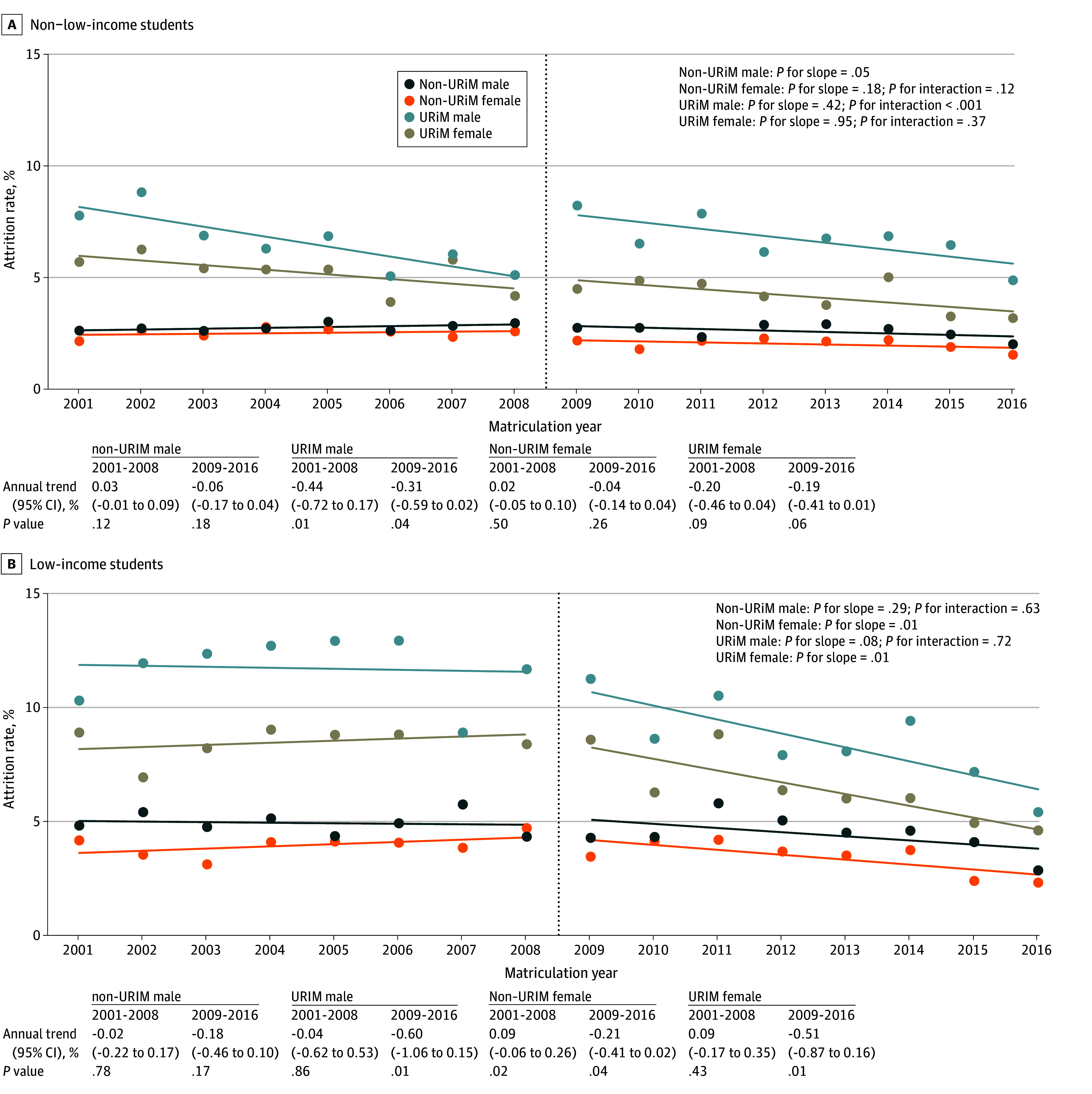
Attrition Rates by Matriculation Year and Intersectional Identities Attrition rates for Doctor of Medicine matriculants from 2001 to 2016 at the intersections of sex, race and ethnicity, and family income. Students self-reported sex, race, and ethnicity on the American Medical College Application Service. URiM indicates individuals underrepresented in medicine (ie, those who identified as Alaska Native, American Indian, Black, Hispanic, Native Hawaiian, or Pacific Islander). American Indian, Alaska Native, Native Hawaiian, and Pacific Islander individuals are grouped together as part of the broader Indigenous community. Dotted lines represent the line of best fit.

Intersectionality analysis shows that after LCME implementation, there were significant differences in annual trend in attrition rates for low-income female students not URiM (2001-2008, −0.04% vs 2009-2016, −0.60%; *P* = .01) and low-income female students who are URiM (2001-2008, 0.09% vs 2009-2016, −0.51%; *P* = .01; Figure 2).

## Discussion

In this cross-sectional study, 2009 LCME diversity standards implementation was associated with a decline in attrition rate, with the largest decline seen for Asian and low-income students. Attrition rates for Black and Hispanic students were declining prior to the 2009 LCME standards and continued with no significant change in annual trend after 2009. Across intersectional groups, the largest decline in attrition rate occurred for low-income female students who are and are not URiM.

The improvement in retention of Asian and low-income students may reflect LCME’s expectations for medical schools to create resources and programs to support diverse learners. Although study results are promising, annual trends in attrition did not significantly improve for Black, Hispanic, and Indigenous students, and the overall attrition rates for these students remain high.^3^ The cross-sectional nature of this study limits inferences of causality between LCME diversity standards and attrition. However, findings may highlight challenges some medical schools face in fostering an inclusive learning environment for historically marginalized students,^4^ emphasizing the need for systematic intervention and holistic student support.^5^ Future studies assessing initiatives at medical schools with low attrition rates are essential to identify effective strategies for retaining diverse learners.

## Notes

Supplement. Data Sharing Statement
