# Supplementary material for: Liaison Committee on Medical Education’s Diversity Standards and Medical School Attrition
Source: JAMA Health Forum. 2025 May 9;6(5):e250697. doi: 10.1001/jamahealthforum.2025.0697 (PMC12065036; doi:10.1001/jamahealthforum.2025.0697)
Supplement: Supplement. — Data Sharing Statement [file jamahealthforum-e250697-s001.pdf]

Data Sharing Statement

Nguyen. Liaison Committee on Medical Education’s Diversity Standards and Medical School Attrition. *JAMA Health Forum*. Published May 09, 2025.
doi:10.1001/jamahealthforum.2025.0697

Data
Data available: No
